# Pharmacodynamic Modeling of Bacillary Elimination Rates and Detection of Bacterial Lipid Bodies in Sputum to Predict and Understand Outcomes in Treatment of Pulmonary Tuberculosis

**DOI:** 10.1093/cid/civ195

**Published:** 2015-03-16

**Authors:** Derek J. Sloan, Henry C. Mwandumba, Natalie J. Garton, Saye H. Khoo, Anthony E. Butterworth, Theresa J. Allain, Robert S. Heyderman, Elizabeth L. Corbett, Mike R. Barer, Geraint R. Davies

**Affiliations:** 1 MalawiLiverpool Wellcome Trust Clinical Research Programme, College of Medicine, University of Malawi, Blantyre; 2 Liverpool Heart and Chest Hospital; 3 Liverpool School of Tropical Medicine, United Kingdom; 4 Department of Microbiology; 5 Department of Medicine, College of Medicine, University of Malawi, Blantyre; 6 Department of Infection, Immunity and Inflammation, University of Leicester; 7 Department of Pharmacology, University of Liverpool; 8 London School of Hygiene and Tropical Medicine; 9 Institute of Infection and Global Health, University of Liverpool, United Kingdom

**Keywords:** tuberculosis, sterilizing activity, persistence, lipid bodies, clinical trials

## Abstract

***Background.*** Antibiotic-tolerant bacterial persistence prevents treatment shortening in drug-susceptible tuberculosis, and accumulation of intracellular lipid bodies has been proposed to identify a persister phenotype of *Mycobacterium tuberculosis* cells. In Malawi, we modeled bacillary elimination rates (BERs) from sputum cultures and calculated the percentage of lipid body–positive acid-fast bacilli (%LB + AFB) on sputum smears. We assessed whether these putative measurements of persistence predict unfavorable outcomes (treatment failure/relapse).

***Methods.*** Adults with pulmonary tuberculosis received standard 6-month therapy. Sputum samples were collected during the first 8 weeks for serial sputum colony counting (SSCC) on agar and time-to positivity (TTP) measurement in mycobacterial growth indicator tubes. BERs were extracted from nonlinear and linear mixed-effects models, respectively, fitted to these datasets. The %LB + AFB counts were assessed by fluorescence microscopy. Patients were followed until 1 year posttreatment. Individual BERs and %LB + AFB counts were related to final outcomes.

***Results.*** One hundred and thirty-three patients (56% HIV coinfected) participated, and 15 unfavorable outcomes were reported. These were inversely associated with faster sterilization phase bacillary elimination from the SSCC model (odds ratio [OR], 0.39; 95% confidence interval [CI], .22–.70) and a faster BER from the TTP model (OR, 0.71; 95% CI, .55–.94). Higher %LB + AFB counts on day 21–28 were recorded in patients who suffered unfavorable final outcomes compared with those who achieved stable cure (*P* = .008).

***Conclusions.*** Modeling BERs predicts final outcome, and high %LB + AFB counts 3–4 weeks into therapy may identify a persister bacterial phenotype. These methods deserve further evaluation as surrogate endpoints for clinical trials.

## Abstract (precis)

Modeling of bacillary elimination from the sputum of patients on tuberculosis therapy predicts long-term outcomes, and identification of intracellular lipid bodies may label antibiotic-tolerant organisms that reduce treatment efficacy. These findings will improve clinical evaluation of new drug regimens.

Tuberculosis treatment takes at least 6 months. Shorter treatment is required [1, 2], particularly in settings of high tuberculosis/human immunodeficiency virus (HIV) coinfection [3]. New compounds are under clinical evaluation [4, 5], but recent phase 3 trials of 4-month regimens incorporating 8-methoxyfluoroquinolones resulted in high relapse rates [6–8] despite promising phase 2 studies [9–12]. New surrogate endpoints, measured early but predicting long-term efficacy, are needed to select better drug combinations.

An obstacle to shorter therapy is bacillary persistence; antibiotic-tolerant organisms without genotypic resistance may survive drug pressure by entering a nonreplicating state [13, 14]. Regimens to kill these bacteria are essential. Modeling bacillary elimination rates (BERs) over 8 weeks may quantitate persistence more precisely than sputum culture conversion at a single time-point and represent better surrogate endpoints [15, 16], particularly if augmented by phenotypic labeling of persister bacteria.

One promising technique is serial sputum colony counting (SSCC). Colony-forming units (CFUs) of *Mycobacterium tuberculosis* are counted after incubation on selective agar and bacillary clearance is measured. Response to first-line therapy is biphasic: a 5- to 7-day early bactericidal phase may represent elimination of metabolically active organisms by isoniazid [17], followed by a sterilization phase lasting many weeks, which represents persister elimination by other drugs. This nonlinear response, and high interindividual variability, necessitates hierarchical mixed-effects modeling of sterilization phase elimination rates (SPERs) to compare novel regimens [12, 18].

An alternative approach involves analyzing serial time-to-positivity (TTP) results in the mycobacterial growth indicator tube (MGIT) system [19, 20]. Liquid cultures remain positive after solid media conversion, which may help study persistence. However, neither SSCC nor MGIT-TTP data are validated to predict relapse.

Identifying antibiotic-tolerant organisms requires single-cell assessment. In vitro studies suggest that nonreplicating bacilli survive physiological stress by upregulating triacylglycerol (TAG) synthase genes, including tgs1, to accumulate cytoplasmic TAG lipid bodies (LBs) [21–23]. Tgs1-expressing organisms demonstrate tolerance to first-line antituberculosis drugs [21, 22], and isoniazid exposure may induce LB formation [24]. LBs may phenotypically label persister cells, but only 1 study has described LB-containing bacilli in clinical samples [23]. Serial analysis during tuberculosis treatment has never been undertaken.

We modeled SSCC and MGIT-TTP culture data from the first 8 weeks of tuberculosis treatment in Malawian adults and performed fluorescence microscopy to quantify LB-positive acid-fast bacilli (%LB + AFB) on sputum smears. We considered whether these putative measurements of persistence predict clinical endpoints.

## METHODS

### Patient Recruitment and Follow-up

A prospective cohort study was conducted at Queen Elizabeth Central Hospital in Blantyre, Malawi, from 2010 to 2012. Consenting adults aged 16–65 years with sputum smear–positive pulmonary tuberculosis graded “++” or “+++” for AFB on Ziehl–Neelsen microscopy were eligible [25]. Exclusion criteria included hemoglobin level <6 g/dL, creatinine level >177 µmol/L, total bilirubin level >51 µmol/L, alanine aminotransferase level >200 IU/L, clinical status suggestive of imminent mortality (World Health Organization Performance Score 4) [26], pregnancy, tuberculosis treatment within 5 years, corticosteroid therapy, or baseline resistance to rifampicin and isoniazid using the Genotype MTBDR*plus* 2.0 line probe assay (LPA; Hain Life Sciences). Treatment was according to National Tuberculosis Control Programme guidelines. Fixed-dose combination tablets containing rifampicin, isoniazid, pyrazinamide, and ethambutol were given for 8 weeks, followed by fixed-dose combination tablets containing rifampicin and isoniazid for 16 weeks [27]. All patients had point-of-care HIV serology. Antiretroviral therapy (ART) was available according to national protocols [28, 29]. Chest radiographs were assessed using a published method [30] to determine the percentage of lung affected and the presence of ≥4-cm cavities.

Follow-up continued for 1 year after end of treatment (EOT). Patients with negative tuberculosis sputum cultures from EOT onward or who stopped coughing and remained well were defined as having stable cure; those with positive culture at EOT were deemed to have failed treatment; and those who were culture negative at EOT but subsequently developed positive cultures were considered to have relapsed.

### Sputum Sample Collection and Processing

Patients were allocated sequentially to staggered, balanced sampling blocks to maximize information for BER modeling [31]. Block 1 submitted sputum on days 0, 4, 14, 28, and 56 of therapy. Block 2 was sampled on days 0, 2, 7, 21, and 49.

Twelve-hour overnight sputum collections were conducted as previously described [18]. Two 1-mL aliquots were used for SSCC plates and liquid cultures. The remainder was stored at −20°C for LB microscopy. Initial sputum processing was done within 24 hours.

All patients submitted spot sputum samples after 5 months of therapy (EOT samples) to assess bacteriological cure. Those with ongoing or recurrent symptoms submitted posttreatment samples to test for relapse.

### Sputum Bacteriology

The SSCC method was previously described [18]. One milliliter of undecontaminated sputum was homogenized with an equal volume of dithiothreitol (Oxoid). Five serial 10-fold dilutions were prepared in phosphate-buffered saline (PBS). Fifty microliters of neat sputum and each dilution was plated onto duplicate plates of Middlebrook 7H11 oleic acid albumin agar media made selective by addition of polymyxin B (200 U/mL), ticarcillin (100 mg/L), trimethoprim (10 mg/L), and AmB (10–30 mg/L). After 3 weeks’ incubation, dilutions yielding 10–100 colonies were selected for counting. Average CFU/mL of sputum were calculated from the 2 replicates.

For liquid culture, 1 mL of specimen was decontaminated with *N*-acetyl-l-cysteine/sodium hydroxide (NaOH) 3% and inoculated into MGITs (Becton Dickinson). TTP recorded by the instrument was used as an inverse measure of bacterial growth. Ziehl–Neelsen microscopy and MGIT TBc Identification Test kits (Becton Dickinson) were used to confirm that positive isolates represented pure growth of *M. tuberculosis*. Samples not signaling positive at 7 weeks were regarded as “negative.”

Sputum samples from EOT and follow-up visits were decontaminated and inoculated on Lowenstein-Jensen media and in MGIT. *Mycobacterium tuberculosis* growth from >1 culture represented failure or relapse, dependent on the timing of the first positive specimen.

### Fluorescence Auramine LipidTOX Red Sputum Microscopy

As fluorescence microscopy to quantitate bacillary subpopulations is difficult on “scanty” slides, baseline assessment was restricted to patients with smear “+++” pretreatment samples. For serial analysis, all samples were reviewed from each patient with an unfavorable outcomes whose sputum was graded “+++” at baseline and at least “++” on 1 or more subsequent occasions. These patients were randomly matched to 3 controls with the same smear grading criteria, but who achieved stable cure. We evaluated changes in the proportion of LB-positive cells, rather than changes in the absolute numbers of AFB.

The Auramine LipidTOX Red method was adapted from an “Auramine-Nile red” technique [32]. LipidTOX Red neutral (LTR; Invitrogen) replaced Nile Red to stain intracellular lipid because Nile Red is solvatochromatic, prone to photo bleaching, and has emission spectrum overlap with auramine O [33, 34]. One milliliter of sputum aliquots was incubated with an equal volume mixture of lipase (1 mg/kg) from *Candida rugosa* (Sigma) and dithiothreitol for 1 hour to digest extracellular lipid and liquefy the sample. Ten-microliter smears were heat-fixed onto slides, flooded with auramine O for 10 minutes, decolorized with 0.5% acid alcohol for 2 minutes, reflooded with LTR (1:200 dilution of stock solution in PBS) for 20 minutes, and counterstained with 0.1% potassium permanganate for 45 seconds. Slides were washed in mycobacteria-free distilled water after each step, protected from light, and read within 24 hours.

Smears were scanned systematically through a fluorescein isothiocyanate filter using a Leica DMLB epifluorescence microscope at ×1000 magnification. All fields containing auramine-stained, yellow-green AFB were photographed using a Leica DFC300FX R2 digital camera, then rephotographed through a tetramethylrhodamine filter to capture LTR-stained red LBs. Smears were examined for 15 minutes or until 100 sequential AFB had been imaged. Organisms on paired images were LB positive if ≥1 LB was seen inside the auramine-labelled bacillus and LB negative if LBs were absent (example images are shown in Supplementary Figure 1*A*–*C*). The %LB + AFB counts were allocated to each slide as follows:

$$\%\mathrm{LB}+\mathrm{AFB}=100\;\left(\frac{\text{Total LB}-\text{positive AFB on all images }}{\text{Total AFB on all images }}\right).$$

Clinical details were blinded before staining. Duplicate smears were made from each specimen and results expressed as mean %LB + AFB counts. Microscopic images were assessed by 2 independent readers.

### Data Analysis and Statistical Methods

The study endpoint was the composite “unfavorable” clinical outcome of treatment failure or relapse. Nonparametric summary statistics were used to describe the data, multivariate logistic regression to assess factors contributing to clinical outcome, and multivariate linear regression to assess factors contributing to bacillary clearance or %LB + AFB counts. Results were expressed as odds ratios (ORs) or regression coefficients with 95% confidence intervals (CIs). Significance was reported at *P* < .05. Interreader variability in %LB-AFB counts was evaluated using Lin concordance coefficient (ρ_c_) and Bland and Altman 95% limits of agreement.

SSCC data modeling employed nonlinear mixed effects (NLME) methods [16, 18, 23]. Patients with ≥2 positive counts were included. Counts below the limit of detection (1.27 log_10_ CFU/mL) were accounted for using a partial likelihood method. Multiexponential functions were fitted to pooled data by nonlinear least squares and compared using the Akaike information criterion and residual plots. The function

$$\log_{10}\text{CFU}=\log_{10}[(e^{\theta 1}\times e^{-\mathrm{day}\;\times \;e^{\theta 2}})+(e^{\theta 3}\times e^{-\mathrm{day}\;\times \;e^{\theta 4}})]$$

was most appropriate and represented the anticipated biphasic pattern of bacillary elimination. Parameters θ_1–4_, expressed on the natural log scale, were transformed to *A*_Int_, α, *B*_Int_, and β on the log_10_ scale for easier evaluation. Supplementary Figure 2*A* interprets the model; the early bactericidal phase is represented by the total baseline bacillary load (*A*_Int_) and early elimination rate constant (α), whereas the sterilization phase is represented by the baseline load of persister organisms (*B*_Int_) and late elimination rate constant (β). Random effects on *A*_Int_, *B*_Int_, and β described interpatient variability. Best linear unbiased estimates of β were extracted for each individual to examine relationships between the SPER and treatment outcome.

MGIT data modeling included patients with ≥2 TTP measurements. A partial likelihood method was used to account for TTP of negative results. Quadratic and spline functions were used to test for curvature in the pooled dataset, but a linear mixed-effects function of the form

TTPindays=a+b(weeksontherapy)

with random effects on *a* and *b* was appropriate. Supplementary Figure 2*B* interprets the intercept (*a*) as a model-derived measurement of baseline TTP and the rate constant (*b*) as the MGIT bacillary elimination rate (MBER). Unbiased MBER estimates were extracted for each patient to assess the relationship between bacillary clearance and treatment outcome.

Analyses were performed in R version 2.15.2 [35]. The M3 method was required to fit the partial likelihood models in NONMEM VII version 2.0 with Pirana (Icon Development Solutions).

### Ethical Approval

Ethical approval was given by the Liverpool School of Tropical Medicine (protocol 09.67) and the College of Medicine Research Ethics Committee, University of Malawi (P.01/10/855).

## RESULTS

### Patients and Samples

**Figure 1.**
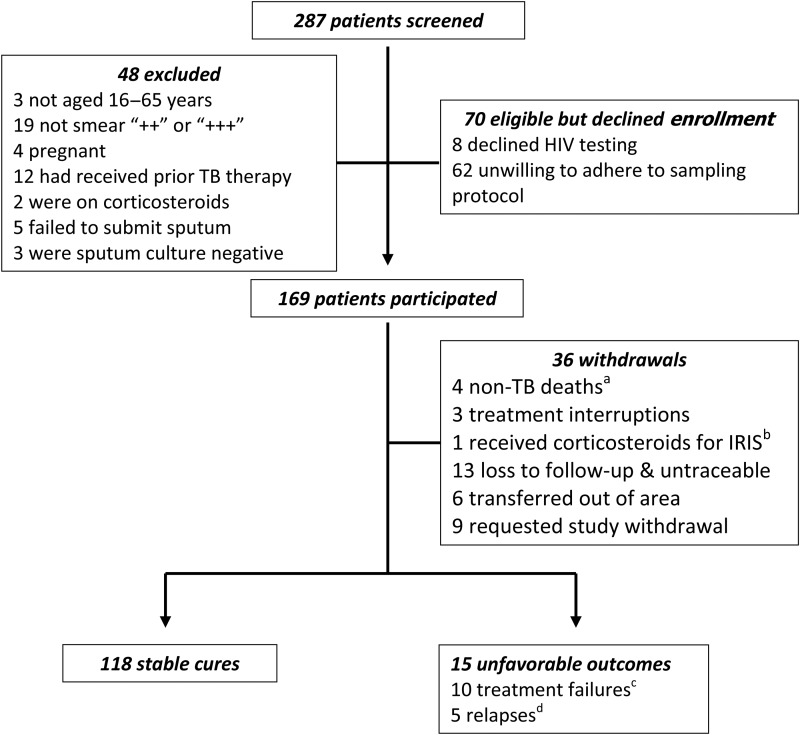
Patient screening, recruitment, and follow-up. ^a^Four patients died after sputum smear and conversion to negative. All were human immunodeficiency virus (HIV) infected and had no ongoing symptoms of active tuberculosis (TB). As the cause of death was not attributed to TB by the study doctor or attending physician, these patients were not deemed to have reached a study endpoint and were withdrawn from the analysis; ^b^Immune reconstitution inflammatory syndrome (IRIS); ^c^One patient died during the second week of therapy while still sputum smear and culture positive for *Mycobacterium tuberculosis*. The cause of death was attributed as TB; ^d^One patient redeveloped a productive cough during posttreatment follow-up, and sputum was smear and culture positive for *M. tuberculosis*. The cause of death was attributed as TB.

Figure 1 describes recruitment, follow-up, and outcomes. Of 287 patients screened, 169 were recruited and 36 withdrew before study completion. Of the remaining 133, there were 118 (89%) stable cures and 15 (11%) unfavorable outcomes (10 failures and 5 relapses, all within 6 months of EOT).

Further analysis was undertaken on patients reaching a study endpoint. Median age was 31 years, and 89 (67%) were male. Median body mass index at baseline was 18.4 kg/m^2^, and 44 (37%) patients had cavities on chest radiograph. Seventy-six (56%) were HIV infected with a median CD4 count of 163 cells/µL. Twenty-five (33%) HIV-infected patients were on ART at recruitment.

Adherence to medication was good; only 3 (2%) patients missed ≥3 doses of tuberculosis treatment at any time. Sixty-four of 76 (85%) HIV-infected patients initiated ART by study end.

LPA analysis confirmed that all patients with unfavorable outcomes harbored drug-susceptible *M. tuberculosis* at baseline. Only 2 acquired resistance thereafter; EOT isolates from 1 treatment failure showed rifampicin (*rpoB*) and isoniazid (*katG*) mutations, and posttreatment isolates from 1 relapse showed an isoniazid (*katG*) mutation.

Table 1 illustrates a trend toward unfavorable outcomes in HIV-infected individuals. No other clinical factor predicted outcome.

**Table 1.** Characteristics and Clinical Outcomes of Study Patients

Characteristic	Total (N = 133)	Unfavorable Outcome (n = 15)	Stable Cure (n = 118)	OR for Unfavorable Outcome (95% CI)	*P* Value
Baseline patient profile					
Male sex	89 (67)	10 (67)	79 (67)	0.99 (.32–3.09)	.983
Age, median (IQR)	31 (26–37)	30 (27–35)	31 (25–38)	1.02 (.96–1.08)	.553
BCG vaccinated	107 (80)	13 (87)	94 (81)	1.51 (.32–7.16)	.607
BMI, kg/m^2^, median (IQR)	18.4 (17.2–20.0)	19.5 (17.8–20.3)	18.3 (17.1–19.9)	1.18 (.96–1.45)	.108
HIV infected	76 (56)	12 (80)	64 (54)	3.49 (.94–13.02)	.063
CD4 count, cells/µL, median (IQR)^a^	168 (104–314)	168 (66–349)	164 (104–301)	1.00 (1.00–1.00)	1
% of lung affected on CXR, median (range)^b^	25 (18–40)	25 (18–38)	25 (18–40)	0.99 (.96–1.03)	.605
Presence of cavity ≥4 cm diameter^b^	44 (37)	4 (31)	41 (38)	0.72 (.21–2.47)	.597
Isoniazid-monoresistant *M. tuberculosis*	2 (2)	0 (0)	2 (2)	NA	NA
Sputum bacillary load, log_10_ CFU/mL, median (IQR)	6.19 (4.94–7.66)	6.56 (5.37–7.59)	6.17 (4.85–7.65)	1.06 (.74–1.52)	.748
Sputum MGIT-TTP, d, median (IQR)	4.00 (3.00–7.50)	3.50 (3.50–6.13)	4.50 (3.00–7.50)	0.94 (.80–1.09)	.405
%LB+AFB count, % median (IQR)^c^	28 (13–44)	22 (13–54)	30 (13–43)	1.00 (.97–1.03)	.927
Progress during TB therapy					
Missed a total of >3 doses of TB therapy	6 (5)	2 (14)	4 (4)	4.75 (.79–28.7)	.090
Rise in BMI by 8 wk, kg/m^2^, median (IQR)	0.72 (0.04–1.34)	0.70 (0.01–1.52)	0.72 (0.11–1.31)	1.03 (.67–1.58)	.908
Rise in BMI by end of follow-up, kg/m^2^, median (IQR)	1.56 (0.94–2.49)	1.57 (0.19–2.26)	1.54 (0.98–2.48)	0.86 (.56–1.31)	.470
Positive sputum smear at 2 mo	22 (18)	1 (8)	21 (19)	0.35 (.04–2.84)	.325
SSCC culture positive at 8 wk^d^	6 (6)	4 (31)	2 (2)	19.33 (3.10–120.63)	.002
MGIT culture positive at 8 wk^d^	31 (28)	6 (46)	25 (26)	2.50 (.77–8.16)	.128
Any positive sputum culture at 8 wk^d^	34 (29)	7 (50)	27 (26)	2.85 (.92–8.88)	.071

Data are presented as No. (%) unless otherwise specified.

Abbreviations: BMI, body mass index; CFU, colony-forming units; CI, confidence interval; CXR, chest radiograph; HIV, human immunodeficiency virus; IQR, interquartile range; %LB+AFB, percentage of lipid body–positive acid-fast bacilli; MGIT, mycobacterial growth indicator tube; NA, not assessed; OR, odds ratio; SSCC, serial sputum colony counting; TB, tuberculosis; TTP, time to positivity.

^a^ Baseline CD4 counts and antiretroviral therapy for HIV-infected patients only.

^b^ CXRs available for 120 patients.

^c^ Baseline %LB+AFB count available for 69 patients.

^d^ Some 8-week SSCC and MGIT results unavailable due to contamination: n = 102 for SSCC, n = 111 for MGIT, and n = 117 for any culture result.

### Evaluation of Bacillary Elimination

#### SSCC Results

**Figure 2.**
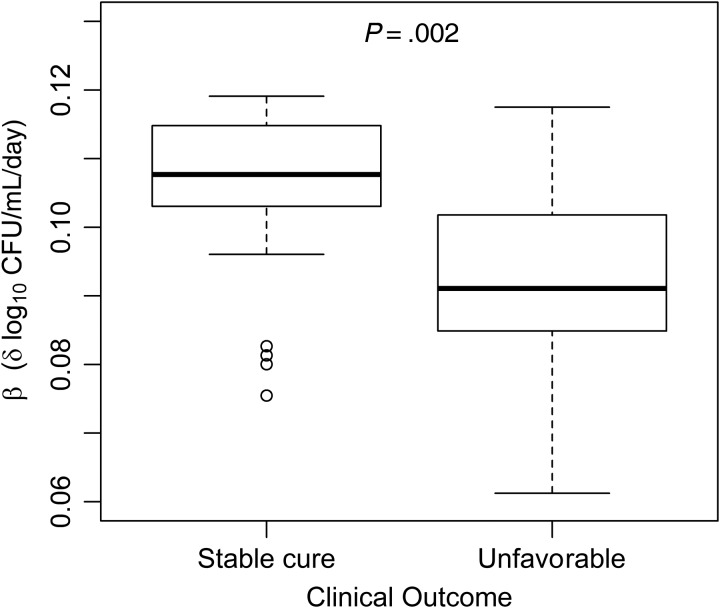
Pharmacodynamic modeling of bacillary elimination by the serial sputum colony counting (SSCC)–nonlinear mixed effects (NLME) method. Using best linear unbiased estimates extracted from the SSCC-NLME model, patients with a higher sterilization phase elimination rate (SPER; β) were less likely to have unfavorable outcomes (odds ratio per 0.01 log_10_ colony-forming units [CFU]/mL/day increase in SPER: 0.39, 95% confidence interval, .22–.70; *P* = .002).

Median baseline bacillary load was 6.19 log_10_ CFU/mL. Six of 102 (6%) patients remained SSCC culture positive at 8 weeks. Baseline variability did not influence outcome, but positive 8-week plates were associated with failure/relapse (OR, 19.33; 95% CI, 3.10–120.63; *P* = .002; Table 1). Rapid culture conversion on solid media and losses to contamination meant that only 86 of 133 (64%) patients contributed sufficient data for inclusion in the SSCC-NLME model. Serial log_10_ CFU/mL counts for these patients and individual bacillary elimination profiles from the bi-exponential model are shown in Supplementary Figures 3*A* and 3*B*. Supplementary Table 1 describes interindividual variability of the SPER (β); a higher baseline log_10_ CFU/mL count was associated with slower SPER. There was a trend toward slower SPER with advancing age, but no relationship with clinical or radiological factors. Figure 2 shows that a slower SPER was strongly associated with unfavorable outcome (OR per 0.01 increase in SPER: 0.39; 95% CI, .22–.70; *P* = .001).

#### MGIT Results

**Figure 3.**
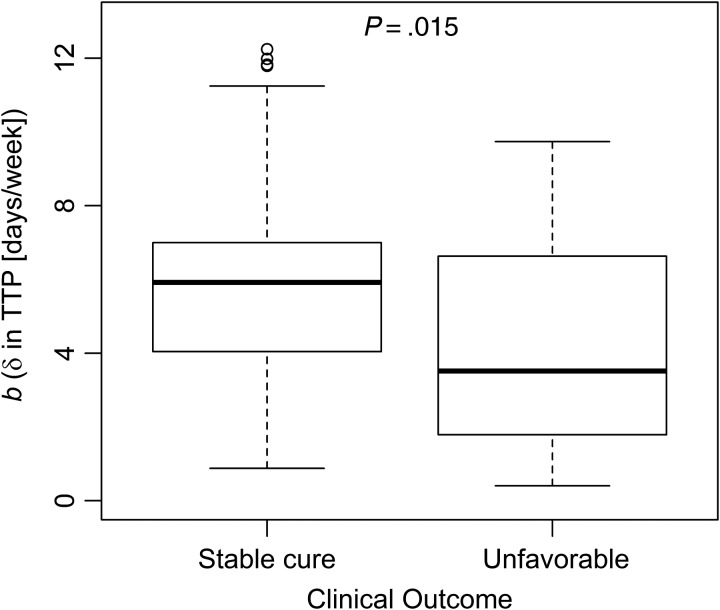
Pharmacodynamic modeling of bacillary elimination by the mycobacterial growth indicator tube linear mixed effects (MGIT-LME) method. Using best linear unbiased estimates extracted from the MGIT-LME model, patients with a higher MGIT bacillary elimination rate (MBER, *b*) were less likely to have unfavorable outcomes (per day of time to positivity [TTP]/week increase in MBER: 0.71; 95% confidence interval, .55–.94; *P* = .015).

Median baseline TTP was 4.0 days. Thirty-one of 111 (28%) patients with 8-week MGIT results remained culture positive. Neither baseline variability nor 8-week culture positivity was associated with unfavorable outcomes. Lower contamination rates, accompanied by greater culture positivity at later time-points, allowed 124 of 133 (93%) patients to contribute data to the MGIT–linear mixed effects model. Serial TTP results and individual bacillary elimination profiles from the model are shown in Supplementary Figures 4*A* and 5*B*. Supplementary Table 2 describes interindividual MBER (*b*) variability; longer baseline TTP was associated with faster MBER, and male sex was associated with slower MBER. Figure 3 shows a strong association between individual MBER estimates and final outcome (OR of unfavorable outcome per 1-day increase in TTP per week of therapy: 0.71; 95% CI, .55–.94; *P* = .015).

### Baseline ALTR Microscopy

Baseline sputum samples were assessed for 69 patients. The median %LB + AFB count was 28%. Two independent observers analyzed each image, and the interindividual concordance coefficient for %LB + AFB counts (ρ_c_) was 0.84 (95% limits of agreement, −29.45%–21.83%). There were no relationships between clinical or radiological factors and baseline %LB + AFB counts.

Ten patients with known baseline %LB + AFB counts had unfavorable outcomes. Fifty-nine had favorable outcomes. Baseline %LB + AFB count was not associated with clinical outcome (Table 1), SPER (Supplementary Table 1), or MBER (Supplementary Table 2).

### Serial ALTR Microscopy

**Figure 4.**
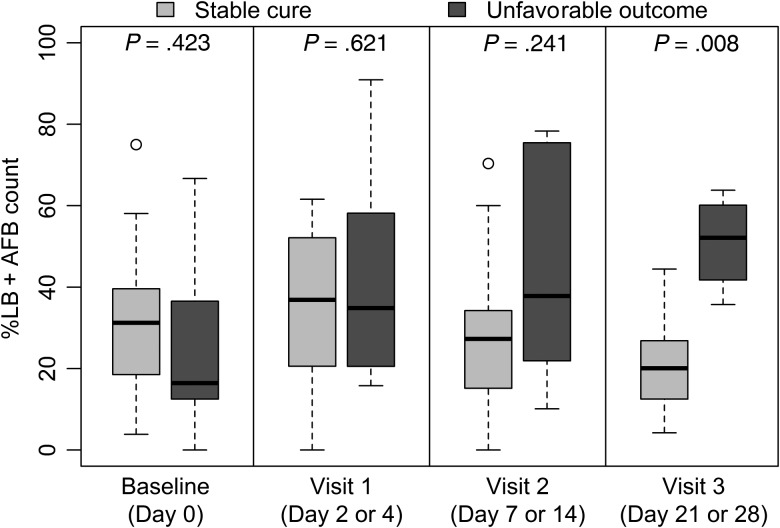
Changes in percentage of lipid body–positive acid-fast bacilli (%LB + AFB) count of serial sputum samples collected from a substudy of patients during tuberculosis therapy. Results are displayed from serial %LB + AFB counts in 38 patients (29 with favorable and 9 with unfavorable outcomes). There were no significant differences in %LB + AFB counts at baseline or during the first 2 treatment visits between patients with different final outcomes. However, the %LB + AFB counts of patients who ultimately had unfavorable outcomes gradually increased during therapy, and by visit 3 (day 21 or 28) were significantly higher than counts in the favorable outcomes groups. Comparisons between groups at visit were made using a Wilcoxon test.

A case-control study of 40 patients (10 with unfavorable outcomes and 30 with favorable outcomes) was undertaken. Images from 1 patient in each group were discarded due to poor-quality smears. Data from 38 patients (9 with unfavorable and 29 with favorable outcomes) were analyzed. Figure 4 illustrates that %LB + AFB counts on smears from each group were similar at baseline and visit 1. By visit 2 there were higher %LB + AFB counts in the unfavorable outcomes group and by visit 3, the difference between groups was significant (*P* = .008). At visit 3, the OR of an unfavorable outcome for each percentage rise in %LB + AFB count was 1.21 (95% CI, .97–1.50; *P* = .088).

## DISCUSSION

The failure of clinical trials to shorten tuberculosis treatment highlights the need for new biomarkers to predict failure/relapse. Our study demonstrates that BER modeling and rising %LB + AFB counts correlate with clinical endpoints.

When relating biomarkers to long-term outcome, posttreatment relapse should ideally be discriminated from reinfection. Unfortunately, in this study, detailed sequencing was unavailable to compare pre- and posttreatment isolates. However, data from Malawi [36] and South Africa [37] suggest that recurrent tuberculosis within 6 months of treatment completion is significantly more likely to be attributable to relapse, even among HIV-infected patients, and our unfavorable outcomes all occurred within this timeframe. LPA analysis indicated that only 2 of 15 patients with unfavorable outcomes acquired isoniazid or rifampicin resistance mutations during therapy, supporting the premise that survival of persister organisms was the main problem.

Sputum culture conversion at 8 weeks is the conventional phase 2 study endpoint, and an association was observed between positive 8-week SSCC plates and unfavorable outcomes. However, 94% of patients (including 73% of failure/relapses) were not SSCC positive at 8 weeks. As a single 8-week data point cannot discriminate between individuals with earlier culture conversion, a large sample would be required to show superiority of any new regimen in a comparative trial [38]. BER models incorporate information from serial samples to describe variation in efficacy more precisely.

The SSCC model was consistent with previous studies [12, 16, 39] and compatible with biphasic bacillary clearance [13]. Our study is the first to describe a relationship between SPERs and individual patient outcomes. Despite technical challenges (eg, contamination [18]), these results argue that SPER estimation is a clinically relevant means of evaluating new tuberculosis treatments.

Positive 8-week MGIT cultures were not associated with unfavorable outcomes, reinforcing observations that phase 2 study results vary on different media [9–12]. A linear rather than bi-exponential model described the MGIT-TTP data, reflecting differences in bacillary populations captured by each microbiology technique. Actively replicating mycobacteria may perish during NaOH processing and are underrepresented in liquid cultures, whereas persisters are more extensively revived from broth [12, 40]. Although a linear model is not divisible into early bactericidal and sterilization phases, the relationship between MBER and outcomes advocates this as an alternative measure of sterilizing activity.

The %LB + AFB counts were studied to test the premise that LBs label persister bacteria. These analyses were performed in patients with high smear-positivity grades because examination of bacterial subpopulations on scanty slides is difficult. Selection of cases with high sputum bacillary loads complicates extrapolation of results to patients with paucibacillary or extrapulmonary disease. However, the observation that higher %LB + AFB counts after 3–4 weeks of treatment correlates with failure/relapse is consistent with in vitro data on antibiotic tolerance [22]. Baseline counts did not affect outcome, suggesting that induction or selection of LB-positive organisms during therapy is more relevant to treatment response. Despite small numbers, these pilot data suggest that study of LB-positive cells may help explain how unfavorable outcomes occur.

There were limitations to this work. It was completed at a single site, so geographical variation in bacillary clearance [15, 41] was not considered. Persister organisms that do not grow on routine media [42] or are non-acid-fast [21, 22, 43] at microscopy may not have been detected. As all patients received the same drugs, additional studies should evaluate how effectively BER modeling compares novel regimens.

Overall, the findings that early quantitation of bacillary persistence predicts the risk of unfavorable outcomes and high %LB + AFB counts during therapy may identify persister bacteria indicate that these methods should be developed as candidate surrogate endpoints for phase 2 studies. If reproduced in other settings, our results could crucially impact the conduct of clinical trials of new tuberculosis regimens.

## Supplementary Data

Supplementary materials are available at *Clinical Infectious Diseases* online (http://cid.oxfordjournals.org). Supplementary materials consist of data provided by the author that are published to benefit the reader. The posted materials are not copyedited. The contents of all supplementary data are the sole responsibility of the authors. Questions or messages regarding errors should be addressed to the author.

Supplementary Data
