# Supplementary material for: Pharmacodynamic Modeling of Bacillary Elimination Rates and Detection of Bacterial Lipid Bodies in Sputum to Predict and Understand Outcomes in Treatment of Pulmonary Tuberculosis
Source: Clin Infect Dis. 2015 Mar 16;61(1):1–8. doi: 10.1093/cid/civ195 (PMC4463005; doi:10.1093/cid/civ195)
Supplement: Supplementary Data [file supp_61_1_1__index.html]

Pharmacodynamic modelling of bacillary elimination rates and detection of bacterial lipid bodies in sputum to predict and understand outcomes in treatment of pulmonary tuberculosis — Pharmacodynamic Modeling of Bacillary Elimination Rates and Detection of Bacterial Lipid Bodies in Sputum to Predict and Understand Outcomes in Treatment of Pulmonary Tuberculosis — Pharmacodynamic Modeling of Bacillary Elimination Rates and Detection of Bacterial Lipid Bodies in Sputum to Predict and Understand Outcomes in Treatment of Pulmonary Tuberculosis — Supplementary Data 

# Pharmacodynamic Modeling of Bacillary Elimination Rates and Detection of Bacterial Lipid Bodies in Sputum to Predict and Understand Outcomes in Treatment of Pulmonary Tuberculosis

## Supplementary Data

Supplementary Data

**Files in this Data Supplement:**

- Supplementary Figures - docx file
- Supplementary Table 1 - docx file
- Supplementary Table 2 - docx file
