# Supplementary material for: Pharmacodynamic Modeling of Bacillary Elimination Rates and Detection of Bacterial Lipid Bodies in Sputum to Predict and Understand Outcomes in Treatment of Pulmonary Tuberculosis
Source: Clin Infect Dis. 2015 Mar 16;61(1):1–8. doi: 10.1093/cid/civ195 (PMC4463005; doi:10.1093/cid/civ195)

**Supplementary Figure 1: Fluorescence microscopy images of ALTR stained sputum smears**

A& B: LB positive organisms in merged FITC and TRITC photographs. Both images show 2 auramine–stained bacilli, both of which containing several LTR labelled 3 LBs. C: LB negative organisms in merged FITC and TRITC images. 3 auramine-stained bacilli are clearly seen, but none contain any LTR labelled LBs

**Supplementary Figure 2: Schematic representation of SCC-NLME and MGIT-LME model functions and parameters**

A: For the SSCC-NLME model, a bi-exponential function fits a biphasic curve to the SSCC data. The first (early bactericidal) phase is represented by the total baseline bacillary load (θ_1_) and an early elimination rate co-efficient (θ_2_). The second (sterilisation) phase is represented by the baseline load of persister organisms (θ_3_) and a late elimination rate co-efficient (θ_4_). θ_1-4_ are expressed on the natural log scale and may be converted to A_Int_, α, B_Int_ and β on the log_10_ scale for clinical interpretation. Calculations for these conversions are shown in the inset box. β is the Sterilisation Phase Elimination Rate (SPER) and is the key pharmacodynamic parameter for assessment of treatment response.

B: For the MGIT-LME model, a simple linear function is fir to the MGIT data. *a* represents baseline TTP, whilst *b* represents the change in TTP as bacilli are killed during TB therapy. *b* is the MGIT Bacillary Elimination Rate (MBER) and is the key pharmacodynamic parameter for assessment of treatment response.

**Supplementary Figure 3: Pharmacodynamic modelling of bacillary elimination by the SSCC-NLME method**

A: log_10_CFU/ml data for all patients included in the SSCC-NLME model. B: After fitting the bi-exponential model by partial likelihood, unbiased parameter estimates for each individual are shown (SC: Patients who achieved stable cure, UO: Patients with unfavourable outcomes). The second rate constant on each profile (β) represents the sterilisation phase elimination rate (SPER). The population fixed effect of the SPER (β) was 0.095 log_10_CFU/ml/day.

**Supplementary Figure 4: Pharmacodynamic modelling of bacillary elimination by the MGIT-LME method**

A: TTP data for all patients included in the MGIT-LME model. B: After fitting the linear model by partial likelihood, unbiased parameter estimates for each individual are shown. The slope of the line on each profile (*b*) represents the MGIT Bacillary Elimination Rate (MBER). The population fixed effect of the MBER (*b*) was 5.9 days/week of therapy.


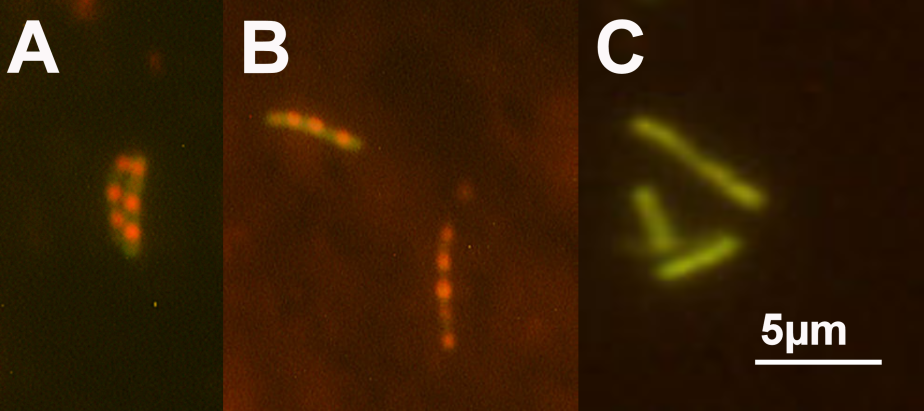


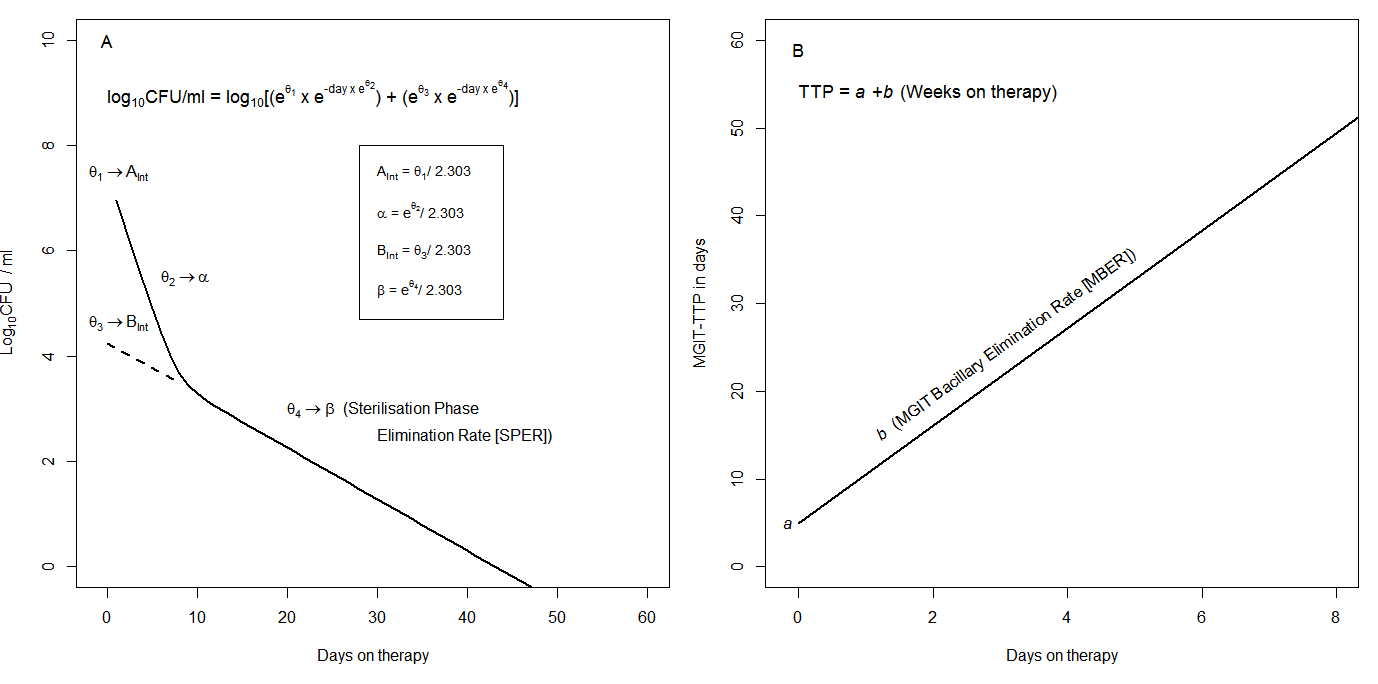


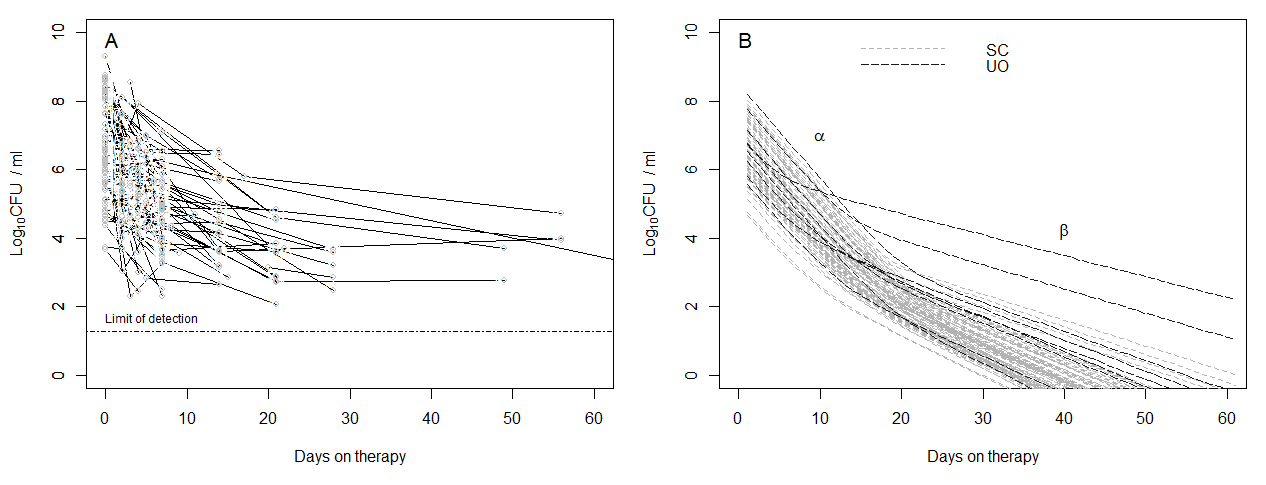


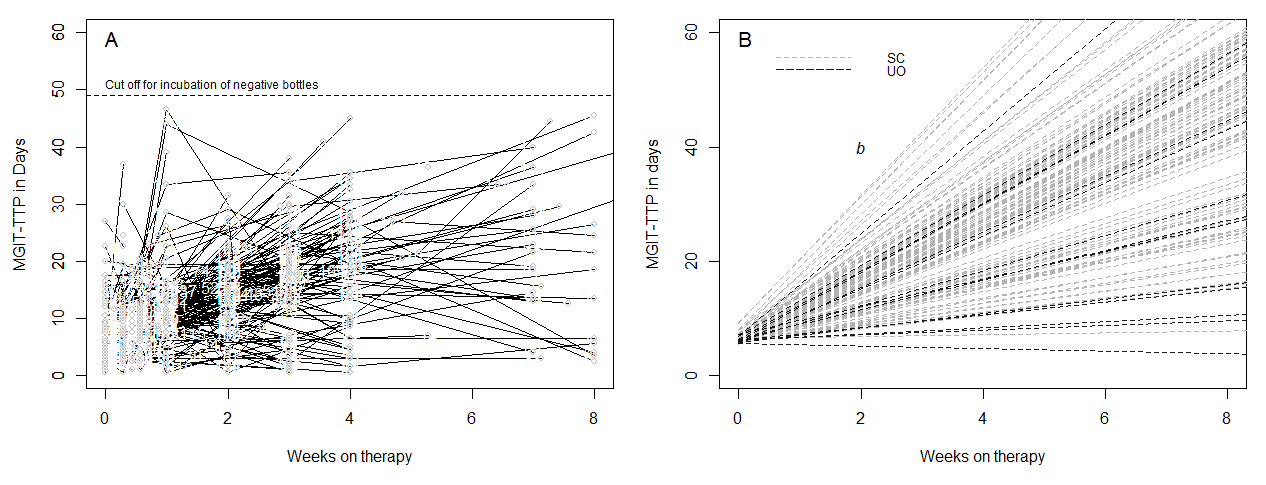

Supplement: Supplementary Data [file supp_civ195_civ195supp_figs.docx]
