# Supplementary material for: Pharmacodynamic Modeling of Bacillary Elimination Rates and Detection of Bacterial Lipid Bodies in Sputum to Predict and Understand Outcomes in Treatment of Pulmonary Tuberculosis
Source: Clin Infect Dis. 2015 Mar 16;61(1):1–8. doi: 10.1093/cid/civ195 (PMC4463005; doi:10.1093/cid/civ195)
Supplement: Supplementary Data [file supp_civ195_civ195supp_table1.docx]

**Supplementary Table 1: Clinical and radiological factors influencing SPER**

|  | **Univariate analysis** | | **Multivariate analysis** | |
| --- | --- | --- | --- | --- |
|  | **Effect on SPER (95% CI)** | **p-value** | **Effect on SPER (95% CI)** | **p-value** |
| Male sex | -1.26 (-6.60 to 4.07) | 0.639 | - | - |
| Age in years | -0.26 (-0.54 to 0.02) | 0.066 | -0.23 (-0.52 to 0.06) | 0.051 |
| BCG vaccinated | -4.77 (-10.63 to 1.08) | 0.109 | - | - |
| Baseline BMI in kg/m^2^ | -0.83 (-1.90 to 0.24) | 0.125 | - | - |
| HIV infected | -2.56 (-7.28 to 2.16) | 0.285 | - | - |
| Baseline CD4 count in cells/µl | -0.02 (-0.04 to 0.01) | 0.216 | - | - |
| % of lung affected on CXR | 005 (-0.11 to 0.20) | 0.559 | - | - |
| Presence of cavity ≥4cm diameter on CXR | 2.16 (-2.89 to 7.20) | 0.398 | - | - |
| Baseline sputum bacillary load in log_10_CFU/ml | -1.87 (-3.30 to -0.44) | 0.011 | -1.68 (-3.12 to -0.24) | 0.022 |
| Baseline %LB+AFB count | -0.02 (-0.19 to 0.15) | 0.784 | - | - |
