# Supplementary material for: Pharmacodynamic Modeling of Bacillary Elimination Rates and Detection of Bacterial Lipid Bodies in Sputum to Predict and Understand Outcomes in Treatment of Pulmonary Tuberculosis
Source: Clin Infect Dis. 2015 Mar 16;61(1):1–8. doi: 10.1093/cid/civ195 (PMC4463005; doi:10.1093/cid/civ195)
Supplement: Supplementary Data [file supp_civ195_civ195supp_table2.docx]

**Supplementary Table 2: Clinical and radiological factors influencing MBER**

|  | **Univariate analysis** | | **Multivariate analysis** | |
| --- | --- | --- | --- | --- |
|  | **Effect on MBER (95% CI)** | **p-value** | **Effect on MBER (95% CI)** | **p-value** |
| Male sex | -1.69 (-2.58 to -0.80) | <0.001 | -1.32 (-2.28 to -0.36) | 0.007 |
| Age | 0.01 (-0.04 to 0.06) | 0.699 | - | - |
| BCG vaccinated | -0.65 (-1.78 to 0.48) | 0.257 | - | - |
| Baseline BMI in kg/m^2^ | 0.10 (-0.01 to 0.29) | 0.292 | - | - |
| HIV infected | -0.09 (-0.97 to 0.80) | 0.848 | - | - |
| Baseline CD4 count in cells/µl | 0.00 (-0.01 to 0.00) | 0.263 | - | - |
| % of lung affected on CXR | -0.04 (-0.07 to -0.01) | 0.005 | - | - |
| Presence of cavity ≥4cm diameter on CXR | -1.02 (-1.95 to -0.10) | 0.031 | -0.19 (-1.11 to 0.74) | 0.691 |
| Baseline sputum MGIT-TTP in days | 0.25 (0.16 to 0.34) | <0.001 | 0.2 (0.10 to 0.29) | <0.001 |
| Baseline %LB+AFB count | -0.01 (-0.04 to 0.01) | 0.257 | - | - |
